# Magnetic skyrmions: intriguing physics and new spintronic device concepts

**DOI:** 10.1093/nsr/nwy109

**Published:** 2018-10-08

**Authors:** Yan Zhou

**Affiliations:** School of Science and Engineering, The Chinese University of Hong Kong, China

The mathematical concept of skyrmions was first introduced by high-energy physicist Tony Skyrme in 3D space for describing the stability of hadrons as objects possessing non-trivial topological numbers in a non-linear sigma model. Later on, the original concept of skyrmions was generalized to various condensed-matter systems such as Bose–Einstein condensates, liquid-crystal phases, photonic systems, and quantum Hall systems exhibiting the quantum Hall effect [1–3]. Recently, skyrmions have been realized in magnetic crystals and multilayers lacking inversion symmetry. Distinct from several topologically protected objects or states such as quantum Hall states, chiral edge states of topological insulators, massless Majorana modes etc., magnetic skyrmions exhibit unique real-space topological characteristics and are envisioned as one of the most promising information carriers in consumer low-power spintronic devices [1,2]. In this perspective, we do not attempt to provide a complete overview of this exciting and fast-growing field, but rather highlight the new topological physics and typical device concepts in the field of skyrmionics, with particular focus on magnetic skyrmions in ultrathin films and multilayers with interfacial Dzyaloshinskii–Moriya interaction (DMI).

Magnetic skyrmions were initially identified in non-centrosymmetric crystals of certain helimagnetic materials, such as MnSi, FeGe, MnFeGe, FeCoSi, etc. [1–3], stabilized by chiral interactions between atomic spins. Later, magnetic skyrmions were also discovered in ultrathin films and multilayers, where they are stabilized by interfacial DMI, which is present due to the broken inversion symmetry combined with strong spin–orbit interaction at the interface [1–3]. In Fig. 1a and b, we show two typical spin-swirling nanometric spin textures with a well-defined topological charge of unity, where the spins at its periphery point in the opposite direction to the spins in the center. Compared to magnetic bubbles with typical sizes of micrometers, stabilized by dipolar interactions, the typical size of a skyrmion stabilized by DMI ranges from 1–100 nm depending on the material parameters, applied magnetic field, and temperature.

**Figure 1.**
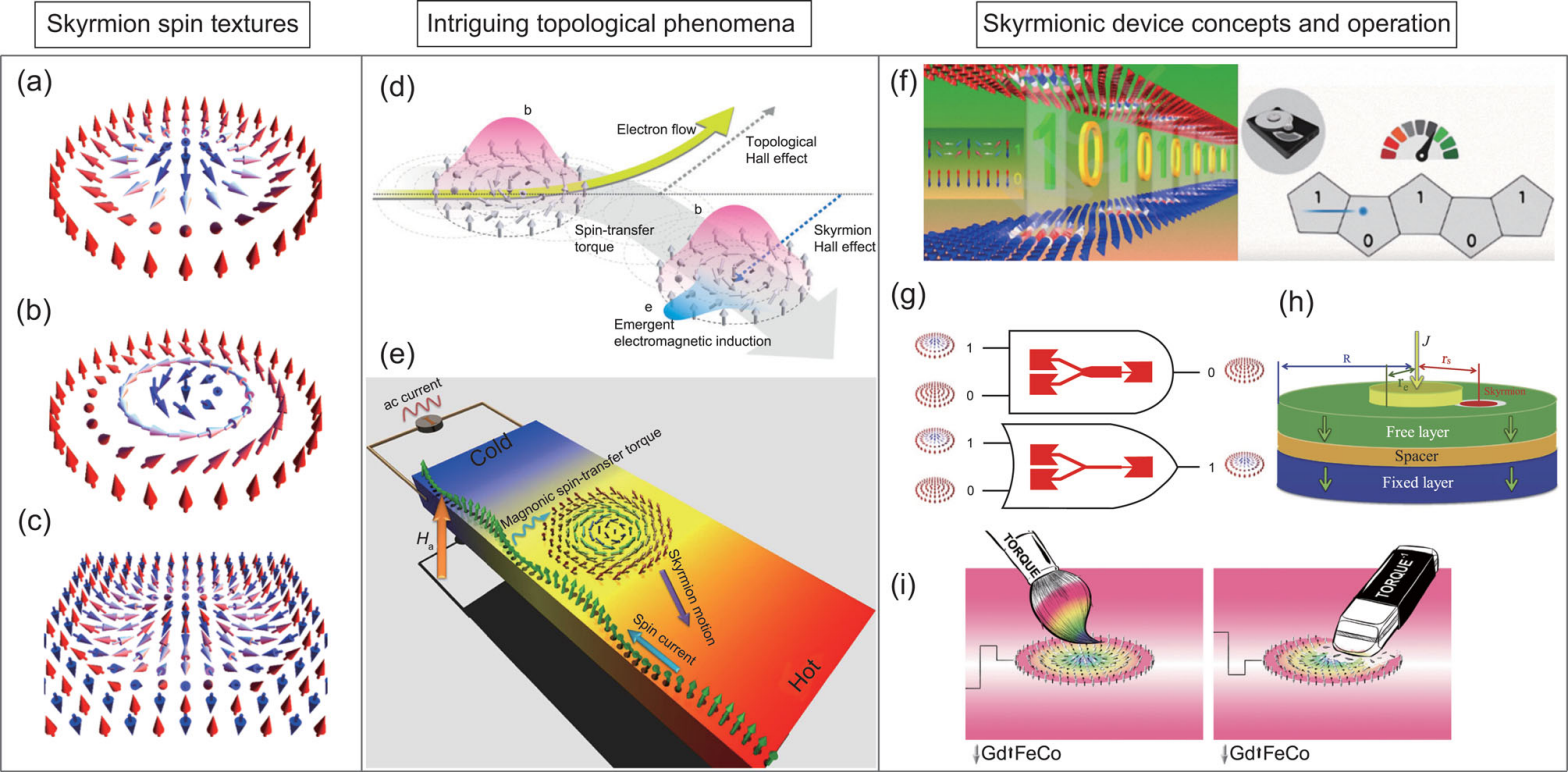
Left panel: Typical skyrmion spin textures where the spin textures are indicated by the arrows. (a) Néel-type skyrmion; (b) Bloch-type skyrmion; (c) antiferromagnetic skyrmion. Middle panel: Typical topological phenomena of skyrmions. (d) Schematic of the topological Hall effect and skyrmion Hall effect; (e) Skyrmion motion driven by a magnon current. Right panel: Typical skyrmionic device concepts. (f) Skyrmion racetrack memory; (g) logic devices; (h) skyrmion oscillator; (i) painting and erasing ferrimagnetic skyrmions. Panel (d) is adapted with permission from [3], Macmillan Publishers Limited. Panel (e) is adapted with permission from [4], American Physical Society. Panel (h) is adapted with permission from [7], Institute of Physics. Panel (i) is adapted with permission from [9,20], Macmillan Publishers Limited.

Magnetic skyrmions exhibit intriguing and novel phenomena due to their topologically non-trivial spin textures. One pertinent example is the topological Hall effect induced by the emergent magnetic field of the skyrmions on conduction electrons and its reciprocal effect, i.e. the skyrmion Hall effect [3] (see Fig. 1d). Analogous to the charge Hall effect, which describes the transverse deflection of electric charges due to the Lorentz force, magnetic skyrmions exhibit curved trajectories away from the direction of applied current as a result of their spin topology. Another intensively researched topic about skyrmion fundamental physics is the interaction between thermally or electrically excited spin waves (magnons) and skyrmions [4] (see Fig. 1e). In addition to being attractive for fundamental research, these fascinating spatially localized nanometric particle-like spin textures can be driven by very small electric currents, magnetic or electric field pulses, thus possessing a huge potential for technological applications. Thanks to their unique spin topology, which allows the skyrmions to avoid pinning potentials created by structural imperfections, the depinning current density of magnetic skyrmions in magnetic crystals could be four to five orders of magnitude smaller than that for driving domain walls in nanowires [1,2]. Magnetic skyrmions are thus envisioned as information carriers for future non-volatile, low power consumption, high-density spintronic memory and logic computing devices. More recently, voltage-gated skyrmion transistor-like functional devices, multiplexers, skyrmion synaptic devices and skyrmionic devices for probabilistic computing applications have been theoretically proposed and some have been experimentally demonstrated [5–10]. Figure 1f–i shows a few typical examples of skyrmionic conceptual devices and the corresponding experimental operation of skyrmionic bits. For practical applications, it is imperative to demonstrate the creation, transportation and annihilation of skyrmions in a controlled fashion. Magnetic skyrmions can be created by local magnetic fields, by electric fields, by electric currents and local heating, etc. [1–3,10]. By utilizing a geometrical constriction, mutual conversion between domain walls and skyrmions has been demonstrated [11], which lays the foundation for a number of conceptual skyrmion-based devices for memory and logic applications [8]. To move skyrmions, one widely discussed method is based on spin-transfer torque or spin–orbit torque. However, the current-driven skyrmion scheme is inevitably accompanied by detrimental effects such as Joule heating, as well as possibility of the skyrmion being repelled or destroyed at the device edges due to the skyrmion Hall effect. To partially or completely suppress the skyrmion Hall effect, a number of different skyrmion-hosting systems have been proposed, which will be discussed later. Although substantial progress has been made in terms of skyrmion nucleation and manipulation, a reliable and all-electrical scheme integrating electric write-in, transmission and read-out of magnetic skyrmions still remains a challenge. Recent works have focused on the electrical read-out of skyrmions by exploiting the characteristic magneto-transport or the topological Hall effect [2].

In addition to ferromagnetic materials, an increasing number of skyrmion-hosting systems, e.g. ferrimagnetic and antiferromagnetic materials (see e.g. Fig. 1c and i), and frustrated magnets, etc., have been either theoretically proposed or experimentally realized [2]. Skyrmions in these materials demonstrate a number of unique features, offering new functionalities and additional degrees of freedom as compared to conventional magnetic skyrmions. For example, the skyrmion Hall effect of antiferromagnetic skyrmions is completely suppressed since spin torques acting on antiferromagnetic skyrmions do not create a Magnus force [5,12]. Furthermore, the mobility and thermal stability of antiferromagnetic skyrmions can be strongly enhanced due to the absence of the gyrotropic force [1,2,5,6,12]. Recently, the deterministic writing and deleting of a single isolated magnetic skyrmion have been experimentally demonstrated in ferrimagnetic materials at room temperature using electric current pulses [9]. All these important achievements make ferrimagnetic and antiferromagnetic skyrmion-hosting materials an attractive basis for next-generation spintronic devices. Ground-state skyrmion lattices have also been reported in material systems without DMI by fabricating vortex-state nanodots on a magnetic thin film with perpendicular magnetic anisotropy such as Co/Pt or Co/Pd [13,14]. In such systems, the artificial skyrmions can be stabilized at room temperature in the absence of an external magnetic field and DMI, which significantly expands the scope of material candidates for skyrmionic research.

Novel skyrmion device concepts such as bio-inspired components and topological quantum computing, etc., are being actively pursued. For skyrmionic devices in which skyrmions are utilized as information carriers, more energy-efficient ways of creating, manipulating and detecting skyrmionic bits at room temperature and under zero applied magnetic field in all-electrical schemes are still being intensively researched. Some practical issues are yet to be addressed. For example, most of the reported sizes of skyrmions in magnetic multilayers are in the range of 100 nm–1 μm, much larger than the skyrmion size in single crystals or monolayers (in the 10 nm range or below). The driving current density is on the order of 10^7^ A/cm^2^, which is also much higher than that in magnetic single crystals. However, rapid advances have been achieved in materials and structure optimization of magnetic multilayers [15–19], which make us optimistic for realization of fully functionalized skyrmionic devices based on the proposed skyrmion device concepts and prototypes in this exciting field.

## References

[1] Fert A , Cros V, Sampaio J. Nat Nanotech 2013; 8: 152–6. 10.1038/nnano.2013.29 23459548

[2] Fert A , Reyren N, Cros V. Nat Rev Mater 2017; 2: 17031.

[3] Nagaosa N , Tokura Y. Nat Nanotech 2013; 8: 899–911. 10.1038/nnano.2013.243 24302027

[4] Lin SZ , Batista CD, Reichhardt C et al. Phys Rev Lett 2014; 112: 187203. 24856718 10.1103/PhysRevLett.112.187203

[5] Zhang X , Zhou Y, Ezawa M. Sci Rep 2016; 6: 24795. 27099125 10.1038/srep24795 PMC4838875

[6] Zhang X , Zhou Y, Ezawa M. *Nat* Commun 2016; 7: 10293. 10.1038/ncomms10293 PMC4735649 26782905

[7] Zhang SF , Wang JB, Zheng Q et al. New J Phys 2015; 17: 023061.

[8] Zhang X , Ezawa M, Zhou Y. Sci Rep 2015; 5: 9400. 25802991 10.1038/srep09400 PMC4371840

[9] Woo S , Song KM, Zhang XC et al. *Nat* Electron 2018; 1: 288–96.

[10] Kang W , Huang YQ, Zhang XC et al. Proc IEEE 2016; 104: 2040–61.

[11] Zhou Y , Ezawa M. Nat Commun 2014; 5: 4652. 25115977 10.1038/ncomms5652

[12] Barker J , Tretiakov OA. Phys Rev Lett 2016; 116: 147203. 27104724 10.1103/PhysRevLett.116.147203

[13] Li J , Tan A, Moon KW et al. Nat Commun 2014; 5: 4704. 25134845 10.1038/ncomms5704

[14] Gilbert DA , Maranville BB, Balk AL et al. Nat Commun 2015; 6: 8462. 26446515 10.1038/ncomms9462 PMC4633628

[15] Maccariello D , Legrand W, Reyren N et al. Nat Nanotech 2018; 13: 233–7. 10.1038/s41565-017-0044-4 29379203

[16] Hsu PJ , Kubetzka A, Finco A et al. Nat Nanotech 2017; 12: 123–6. 10.1038/nnano.2016.234 27819694

[17] Buttner F , Lemesh I, Schneider M et al. Nat Nanotech 2017; 12: 1040–4. 10.1038/nnano.2017.178 28967891

[18] Litzius K , Lemesh I, Kruger B et al. Nat Phys 2017; 13: 170–5.

[19] Soumyanarayanan A , Raju M, Oyarce ALG et al. Nat Mater 2017; 16: 898–904. 28714983 10.1038/nmat4934

[20] Everschor-Sitte K , Sinova J, Abanov A. Nat Electron 2018; 1: 266–7.

